# Effect of phosphatidylcholine on *pld* gene expression level of *Aspergillus fumigatus* by the real time PCR method and investigations of these genes using bioinformatics analysis

**Published:** 2012-09

**Authors:** AD Noodeh, N Singh, GD Robson

**Affiliations:** 1 Department of Life Sciences, Faculty of Science and Technology, Anglia Ruskin University, Cambridge, UK; 2 Department of Immunology and Microbiology, Faculty of Life Sciences, University of Manchester, Manchester, UK

**Keywords:** *Aspergillus fumigatus*, phospholipase D (PLD), pld gene, real time PCR, gene expression, phospholipid, relationship tree

## Abstract

**Background and Objectives:**

Phospholipases are a group of enzymes that breakdown phospholipid molecules producing second products. These second products play a diverse role in the cell such as signal transduction and digestion in humans. In this study, the effect of phospholipids on the expression of *pld* genes of *A. fumigatus* was investigated. The *pld* genes of this fungus were also investigated using bioinformatics studies.

**Materials and Methods:**

Real-time PCR was performed to study the expression of *pld* genes. These genes were investigated using bioinformatics studies.

**Results:**

There was more significant expression for all three *pld* genes when *A. fumigatus* was grown in the presence of phospholipids in the medium. The sequence of *pld* genes of *A. fumigatus* was also interrogated using bioinformatics analysis and their relationship with the other microorganisms was investigated. The fungal *pld* genes were more closely related to *pld* genes from animals and least related to bacterial *pld* genes.

**Conclusion:**

*afpld*1*, afpld2* and *afpld3* are expressed and are up-regulated by phosphatidylcholine. Although indirect evidence of extracellular PLD activity in *A. fumigatus* was demonstrated, conclusive proof by partially sequencing the isolated protein will be needed and its significance in pathogenicity will have to be assessed by constructing a knockout strain and testing its virulence in a mouse model.

## INTRODUCTION

One of the most prevalent opportunistic human fungal pathogens is *A. fumigatus*. The most serious form of invasive aspergillosis has been found in 4% of all dying patients in a modern European teaching hospital (1). *A. fumigatus* is a naturally thermo tolerant, saprophytic fungus which is frequently found on a wide variety of dead organic material (2). The conidia of *A. fumigatus* are one of the most ubiquitous in the atmosphere (3). The conidia range from 2.5 – 3 µm in diameter and because of their small size they are able to enter the alveoli of the lung (2, 4). Phospholipase D (PLD) acts mainly on choline-containing phospholipids and leads to the release of choline and phosphatidic acid as hydrolysis products. In mammalian cells, two isoforms of PLD have been identified while up to three isoforms have been identified in plants (5–7). To date, most of the PLD activity described in fungi has been intracellular (8). *Pld* genes of this fungus regulate their internalization into the lung epithelial cells and suggest their role in pathogenicity (2).

## MATERIALS AND METHODS

### Strain, media and culture condition

*A. fumigatus* (ATCC 90240) were cultured on Vogels (Vogel 1956) chloramphenicol agar at 37^°^ C up to 24 h with constant shaking (200 rpm) containing 1% (w/v) glucose with or without 0.5% (w/v) phosphatidylcholine (Sigma).

Spore suspension was serially diluted to 10^-4^, 10^-5^ and 10^-6^ spores / ml^-1^ and plated into Petri dishes and incubated overnight at 37°C. For liquid cultures, 50 ml of Vogel's media with or without of phospholipid, were distributed into 250 ml Erlenmeyer flasks and inoculated with 0.1 ml of a 1x 10^8^/ ml^-1^ spore suspension and incubated with shaking (250 rpm) at 37°C up to 24 h.

### RNA extraction and Primers for *afpld* genes

The RNA extracted by RNeasy Mini Kit from Qiagen.

***pld1*:**

5″-GATATCGCCGAGCATTTTGT Tm = 55.3°C

5″-AAATTCCACTGCTCCAATCG Tm = 55.3°C

***pld2*:**

5″-TCCAAGGTCAGGTTTTGGAG Tm = 57.3°C

5″-CTCCATCCCAATATCGCAGT Tm = 57.3°C

***pld3*:**

5″-TGAGAAGATGTTGCGGAGTG Tm = 57.3°C

5″-GGCGCACCATGAGAAATTAT Tm = 55.3°C

### Expression of *afplds* and cDNA synthesis

iQSYBER Green Kit (from BioRad) was utilized for making cDNA from RNA and the real-time PCR reactions were prepared according to the manufacturer's protocol. β-tubulin was used as reference gene. cDNA was made using cDNA Kit from Qiagen.

### Comparative C_T_ method, statistical and bioinformatics analysis

The comparative CT method was used to measure the level expression of the target gene by real-time PCR. CT values for gene are normalised against *β- tubulin* (reference housekeeping gene) to give the normalised ΔC_T_ value.

ΔC_T_ = C_T__Target_ – C_T__Reference_

Standard deviation for the ΔC_T_ values were calculated using the following equation: (9)

SDΔC_T_ = (SD C_T__Target_^2^ – SD C_T__Reference_^2^)^1/2^

To compare the relative expression of a gene grown on phosphatidylcholine compared to absence of phosphatidylcholine, the ΔC_T_ value of the gene grown on lecithin is first subtracted from the ΔC_T_ value of the gene grown on absence of lecithin to give the ΔΔC_T_ value.

ΔΔC_T_ = ΔCT _absence_ _of_ phosphatidylcholine - ΔC_T_ phosphatidylcholine

The fold difference in gene expression when grown on phosphatidylcholine compared to absence of phosphatidylcholine is then calculated using the formula below: (9).

Fold change in gene expression = 2 ^ΔΔC_T_^

As the ΔC_T_ phosphatidylcholine value is subtracted from the ΔC_T_ absence of phosphatidylcholine value, the standard deviation of ΔΔC_T_ is the same as the standard deviation of ΔC_T_ absence of phosphatidylcholine.

For comparing the statistical significance of the fold changes, the student t test was used to compare the ΔC_T_ absence of phosphatidylcholine and ΔC_T_ phosphatidylcholine values of each gene.

DNA sequences were submitted for analysis through the Basic Local Alignment Search Tool (BLAST). BLAST was accessed through the National Centre for Biotechnology Information (NCBI) or through the Bioinformatics Centre, University of Kyoto and used to search for sequence similarities within sequences held on the BLAST database.

## RESULTS

### Bioinformatics analysis of the TIGR *Aspergillus fumigatus* database for *pld* homologues

At the time this work was conducted, the unannotated genomic DNA sequence of *A. fumigatus* from the TIGR institute was publicly available as a series of unassembled variable lengths of contiguous DNA through the TIGR website which could be searched with basic Blast programs. While some fungal sequences and reports of *pld* activity were published or present in public databases, all were intracellular although there was some evidence of a secreted extracellular PLD activity in the culture supernatant from *A. fumigatus* (2). In addition, a partial *pld* gene had previously been identified from *A. fumigatus* (10). The aim of this work was to identify gene(s) encoding *pld* in the genome of *A. fumigatus* using bioinformatics and to make a comparison with orthologues from other published genomes (11).

Using PCR based on conserved regions of *pld* from other fungi, a partial homologue had previously been identified in *A. fumigatus* (12). The translated amino acid sequence of this homologue was used in a number of blast search using NCBI and the TIGR *A. fumigatus* genomic database. Three gene sequences were identified (Table 1).

**Table 1:** The properties of three putative *pld* Genes identified from the *A. fumigatus* genomic DNA.

	*Pld1* (Accession No: Q4WGM8)	*Pld2* (Accession No: Q4WZL4)	*Pld3* (Accession No:Q4WWF3)
Genomic length (bp)	2,936	4,249	5,421
CDS length (bp)	2,640	3,159	5,421
Number of introns	6	4	0
Amino acid length	879	1,053	1,807
Mw (Kd)	101.1	118.6	204.9
PI	5.53	6.28	6.99
Pre-pro splice site	NA	LTA/CE	NA
% identity to *pld1*	NA	43.6	39.8
% identity to *pld2*	43.6	NA	29.7
% identity to *pld3*	39.8	29.7	NA

### Comparison of *pld* Genes from fungi and other organisms

**Fig. 1:**
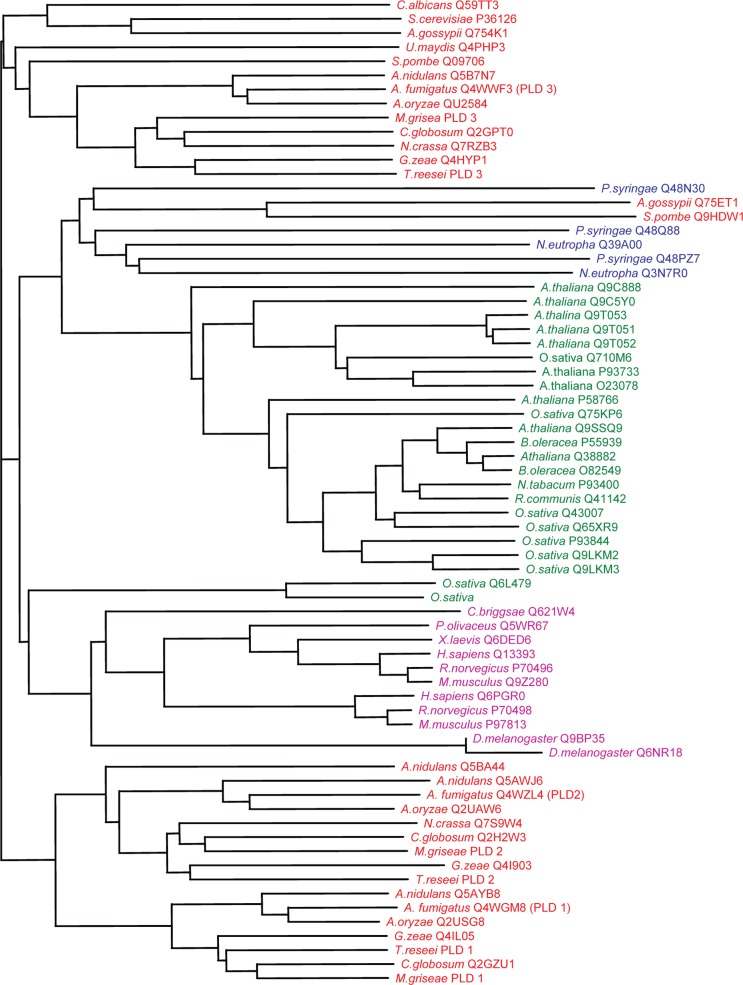
Phylogenetic relationship between the protein sequences of *pld* s from fungi, plants, animals and bacteria

A phylogenetic analysis of all available *pld* protein sequences from bacteria, plants, animals along with derived sequences for the fungi was constructed using ClustalW version 1.7 and a phenogram created by the nearest joining method available from the European Molecular Biology Network (Fig. 1). With the exception of the two *pld*s from *A. gossypii* and *S. pombe*, the fungal *pld*s were more closely related to *pld*s from animals and least related to bacterial *pld*s.

. Fungal *pld*'s are in red, plant *pld*'s in green, bacterial *pld*'s in blue and animal *pld*'s in purple. Codes represent SwissProt accession numbers. *T. reesei* and *M. griseae* were derived from genome sequence and no accession number was available.

### Expression of *pld* genes by real-time PCR

Expression of *pld genes of A. fumigatus* were measured by real time PCR using cDNA and primers which mentioned in section (Table 2). The expression was determined using mRNA from *A. fumigatus* mid-log phase cultures grown at 37°C. Results are pooled from two independent experiments each with at least five replicates normalized to β-tubulin.

**Table 2:** Influence of phosphatidylcholine on the expression levels of *pld* genes of *A. fumigatus*.

Gene	CT without phospholipids factor	ΔCT without phospholipids factor	CT with phospholipids	ΔCT with phospholipids	ΔΔCT	Fold change
*Pld1*	39	18	33	12	6	64●
*Pld2*	27	6	21	0	6	64●
*Pld3*	28	7	23	2	6	32●

● Significant (P < 0.05) change in gene expression (t-test)

## DISCUSSION

Real-time PCR is being increasingly employed to quantify levels of gene expression in a range of fungi (13–16). As shown in Table 2, *afpld*1*, afpld2* and *afpld3* are expressed and are up-regulated by phosphatidylcholine.

As the main site of infection of *A. fumigatus* is through the inhalation of spores which lodge in the lung and are therefore exposed to a phospholipid rich environment, it would appear that many of the extracellular phospholipases are likely to be up-regulated following inhalation.

However, real time PCR on infected lung tissue will need to be performed to confirm if any up-regulation *in vitro*. *A. fumigatus* also secretes PLD (17, 18) and these may be of great significance in lung colonisation.

PLD is known to be a major pathogenic factor in some bacterial pathogens where it causes cell lysis and tissue damage (19). In filamentous fungi, it is usually the case that genes encoding extracellular hydrolyses are normally not expressed or expressed at low levels in the absence of the substrate (20, 21). However, in this case *pld1, pld2, and pld3* were all expressed at relatively high levels in the presence of phospholipid.

Although more investigations on the *pld* genes in the pathogenicity of *A. fumigatus* are needed, a previous study examining a range of *A. fumigatus* isolated from patients and the environment showed that clinical isolates produced significantly higher levels of PLC compared to environmental isolates (18).

PLD was first discovered in plants as an enzyme with phospholipid-specific phosphodiesterase activity, hydrolysing phosphatidylcholine to phosphatidic acid and choline (22). In higher eukaryotes, studies have shown that this enzyme has a rapid activation in response to extracellular stimuli and numerous studies have demonstrated that some PLD's are ‘signal-activated’ phospholipases (22).

PLD is a phospholipid degrading enzyme that generates biologically active products, principally phosphatidic acid which play important functions in cell regulation. In common with many esterases, in addition to hydrolysis, PLD also catalyses a trans-esterification reaction, utilizing short-chain primary alcohols as phosphatidyl-group acceptors. (22). A number of lipid-derived molecules regulate cell proliferation and cell differentiation and specific bioactive lipids serve both as extracellular messengers on cell-surface receptors and as intracellular messengers (23). These messengers are derived from lipid constituents of biological membranes by a number of signal-activated enzymes including phospholipases, lipid kinases, and acylases. These enzymes are activated in response to receptor occupancy (24).

PLD hydrolyses phosphatidylcholine, phosphatidylethanolamine, and phosphatidylinositol to form phosphatidic acid and the head group of the phospholipid substrate (25). Surveys have shown that PLD is regulated by several mechanisms *in vivo* (26).

Intracellular PLD activity has many cellular roles including vesicle formation, protein transport, signal transduction and mitosis (27). Phosphatidic acid, released as a result of PLD activity is itself a second messenger implicated in a variety of cellular events and signal pathway activation (27). In the fungi, PLD is required for the dimorphic transition from the yeast to the hyphal form in *C. albicans* and is required for mitosis in *S. cerevisiae* (27). In *Cryptococcus neoformans*, the cause of cryptococcal meningitis, there is a correlation between PLD production and the size of the capsule in strains isolated from AIDS patients (17). In the present study, the expression level of *pld* genes of *A. fumigatus* was investigated using quantifiable PCR.

In this project, a partial *pld* sequence which had been cloned previously by degenerate PCR (10) was used to investigate the *A. fumigatus* genome sequence in the TIGR database and three *pld* genes were identified and were translated after identifying putative introns using Gene finder software optimised for *A. niger*. All three translated sequences showed significant identity to yeast *pld* and contained two highly conserved *pld* active site motifs. In addition, *Afpld 3* contained a phox-like domain in the N-terminal region which in other eukaryotic *pld's* is thought to interact with phosphoinositides in the membrane and to be involved with cell signalling (12, 28).

When fungal *pld's* were subjected to phylogenetic analysis, *Afpld* 3 and its orthologues grouped separately from *Afpld* 1 and *Afpld* 2 and their orthologues which themselves formed two sub-groups. Thus *Afpld* 1 and *Afpld* 2may have arisen through a gene duplication event. Such gene duplications are commonplace in eukaryotes and are thought to play a pivotal role in the evolution of gene families (29, 30).

Of the eukaryotic genes analysed, only *A. fumigatus pld2* appeared to encode a signal peptide. Some bacterial PLD's are known to be secreted and play a role in pathogenesis. For example, *Neisseria gonorrhoeae*, a strict human pathogen, secretes a *pld* that is involved in membrane ruffling and is required for adhesion and cellular invasion (31). Similarly, extracellular *pld*'s have also been shown to be involved in the virulence of *Yersinia pestis* and *Corynebacterium pseudotuberculosis* (32–34). Phosphatidic acid release through the action of secreted PLD in the lung, may account for some of the inflammatory responses observed following infection (4, 35, 36) as phosphatidic acid is known to act as a pro-inflammatory molecule stimulating the release of inflammatory cytokines (37–39).

In a study Li et al. (2012) showed PLD enzymes facilitate the invasion of *Aspergills fumigatus* to the lung tissue. They showed that the disruption of *pld* genes encoding PLD in *A. fumigatus* significantly decrease intracellular and extracellular phospholipase D activity (40). However, although indirect evidence of extracellular PLD activity in *A. fumigatus* was demonstrated, conclusive proof by partially sequencing the isolated protein will be needed and its significance in pathogenicity will have to be assessed by constructing a knockout strain and testing its virulence in a mouse model.
